# Single-cell transcriptome analysis reveals a cellular immune response in freshwater dark sleeper (*Odontobutis potamophila*) after infection with *Aeromonas veronii*

**DOI:** 10.3389/fphys.2023.1201914

**Published:** 2023-05-18

**Authors:** Guoxing Liu, Chenxi Zhu, Xiaojian Gao, You Zheng, Xinhai Zhu, Hucheng Jiang, Wanhong Wei, Qichen Jiang, Xiaojun Zhang

**Affiliations:** ^1^ College of Animal Science and Technology, Yangzhou University, Yangzhou, China; ^2^ Freshwater Fisheries Research Institute of Jiangsu Province, Nanjing, China; ^3^ Low-temperature Germplasm Bank of Important Economic Fish (Freshwater Fisheries Research Institute of Jiangsu Province) of Jiangsu Provincial Science and Technology Resources (Agricultural Germplasm Resources) Coordination Service Platform, Nanjing, China

**Keywords:** *Aeromonas* veronii, ScRNA-seq, Odontobutis potamophila, immune response, granulocytes, B cell

## Abstract

The bacterium *Aeromonas veronii* is a co-pathogenic species that can negatively impact the health of both humans and aquatic animals. In this study, we used single-cell transcriptome analysis (scRNA-seq) to investigate the effects of infection with *A. veronii* on head kidney cells and the regulation of gene expression in the dark sleeper (*Odontobutis potamophila*). scRNA-seq was used to assess the effects of infection with *A. veronii* in *O. potamophila* B cells, endothelial cells, macrophages, and granulocytes, and differential enrichment analysis of gene expression in B cells and granulocytes was performed. The analyses revealed a significant increase in neutrophils and decrease in eosinophils in granulocytes infected with *A. veronii*. Activation of neutrophils enhanced ribosome biogenesis by up-regulating the expression of *RPS12* and *RPL12* to fight against invading pathogens. Crucial pro-inflammatory mediators *IL1B, IGHV1-4*, and the major histocompatibility class II genes *MHC2A* and *MHC2DAB*, which are involved in virulence processes, were upregulated, suggesting that *A. veronii* activates an immune response that presents antigens and activates immunoglobulin receptors in B cells. These cellular immune responses triggered by infection with *A. veronii* enriched the available scRNA-seq data for teleosts, and these results are important for understanding the evolution of cellular immune defense and functional differentiation of head kidney cells.

## 1 Introduction

*Aeromonas veronii* is a globally distributed pathogenic bacterium that can cause various diseases and affect the healthy growth of aquatic organisms such as fish, shrimp, and shellfish, resulting in huge losses to the aquaculture industry (Hickman-Brenner et al., 1987). *A. veronii* is widely dispersed in rivers, lakes, ponds, and seas and has a high degree of environmental adaptability. It is a typical human-animal-aquatic pathogen that may be isolated from water sources, soil, and the bodies of both humans and animals (Wu et al., 2007).

The dark sleeper (*Odontobutis potamophila*) is a freshwater fish popular in China (Iwata et al., 1985). It has high meat content, tasty flavor, high nutritional value, and excellent health benefits (Zhu et al., 2022). However, in May 2021, *O. potamophila* in a fish farm in Changshu, Jiangsu Province, China, experienced an illness, with skin ulcers as one of the primary symptoms. *Aeromonas veronii* was later shown to be the primary pathogen in the sick fish. Studies have shown that *A. veronii* can cause hemorrhagic septicemia in carp (*Carassius gibelio*) (Sun et al., 2016), tilapia (*Oreochromis niloticus*) (Dong et al., 2017), bass (*L. maculatus*) (Wang et al., 2021), and channel catfish (*Ictalurus punctatus*) (Hoai et al., 2019), mainly manifesting as hemorrhage and congestion of the body surface and organs to varying degrees (Yu et al., 2010). In a prior study of the pathogenicity and histopathology of *A. veronii* in *O. potamophila*, we discovered that *A. veronii* triggered innate immunity and led to mass mortality of the hosts (Liu et al., 2022). Acute mortality of the catfish *Ictalurus lunetas* also occurred after infection by *A. veronii* (Zhang et al., 2016). In *Lateolabrax maculatus*, *A. veronii* infection rapidly activated the chemokine signal pathway and stimulated an acute inflammatory response (Wang et al., 2022).

Molecular understanding of fish immunology is growing, but *in vitro* and *in vivo* research on fish immune activity is still in its infancy. Currently, data on markers for specific fish cell populations and cell subpopulation determinants are limited (Huang et al., 2021), which is an ongoing issue for fish immunologists. However, several cutting-edge methods, including single-cell RNA sequencing (scRNA-seq), are now being used to investigate the cellular immunological functions of teleost fish.

In the present study, we performed scRNA-seq on head kidney cells of *O. potamophila* to characterize the functional heterogeneity of cells. We identified genetic markers for each cell cluster and analyzed their main functions, thereby filling a gap in the taxonomic identification of *O. potamophila* cells. We also comprehensively analyzed the cellular immune response and gene expression profile under the influence of *A. veronii* infection, which is important for a better understanding of the immune response of *O. potamophila* to pathogens.

## 2 Materials and methods

### 2.1 Experimental fish and *A. Veronii* strains

Healthy *O. potamophila* (15 ± 1.5 g) were provided by Yangzhong Base of the Freshwater Fisheries Research Institute of Jiangsu Province, China. *Aeromonas veronii* stl3-1 was isolated from diseased *O. potamophila* (see (Liu et al., 2022) for specific information about the diseased strain). *Aeromonas veronii* stl3-1 was inoculated in a common broth medium, incubated at 28°C and 1,180 g on a shaker for 18 h, centrifuged at 5,000 *g* for 10 min, and the supernatant was discarded. The bacteria in the pellet were resuspended in sterile phosphate-buffered saline at pH 7.4, and the concentration was adjusted to 1.8 × 10^6^ CFU/mL.

### 2.2 Artificial infection experiment

After 1 week of acclimation, healthy *O. potamophila* were divided into an infected group (TAV) and an uninfected control group (CK). Three replicates, each containing 20 fishes, were set up for each group. Each fish in the infected group was injected intraperitoneally with 100 μL (1.8 × 10^6^ CFU/mL) of *A. veronii* stl3-1 suspension. The fish in the control group were injected with sterile phosphate buffered saline (pH 7.4) in the same manner and at the same dose. Fish were sacrificed, and head kidney tissues from the infected and control groups were taken 24 h after injection. To avoid small sample size and individual differences, three head kidney tissues from each biological replicate were mixed to generate a sample).

### 2.3 Ethical statement

All treatments of fish in this study were strictly in accordance with the guidelines of Animal Experiment Ethics Committee of Yangzhou University. The protocol was approved by Animal Experiment Ethics Committee of Yangzhou University (permit number: 201802003).

### 2.3 Cell range analysis and quality control based on full-length transcriptome data

We compared full-length transcripts produced by triple sequencing splicing and performed data quality statistics on the raw data using the 10 × single-cell transcriptome quality control analysis program Cell Ranger (V6.1.2) (Melsted et al., 2021). Single-cell cDNA libraries were sequenced using the double-end sequencing mode of the Illumina HiSeq 4000 sequencing platform. The program locates cell-specific barcode sequence markers in the sequence and unique molecular identifier markers for various mRNA molecules inside each cell to quantify the high-throughput single-cell transcriptome.

### 2.4 Dimensionality reduction and cluster analysis

The filtered data were normalized before analysis by dividing the count value by 10,000 to obtain the log value. We selected the top 2000 highly variable genes for subsequent descending and clustering analysis. Principal component analysis was used for dimensionality reduction, and then Uniform Manifold Approximation and Projection (UMAP) and t-Distributed Stochastic Neighbor Embedding (tSNE) were used for secondary dimensionality reduction and visualization. After the clustering results were obtained, differential gene analysis was performed on different clusters (i.e., screening for marker genes). Marker gene screening criteria were │logFC │> 0.25 and *p* < 0.01. The top 20 highly variable genes were used for the heatmap display which was used to help identify core marker genes. Cells in different clusters of samples from different tissue sources were counted. Barplots were used to display the results and help identify the differential clusters. The Chi-square test was performed for cells in the different grouping of clusters.

### 2.5 Cell subpopulation identification

Since *O. potamophila* were not single-cell annotated, we first compared the transcripts to the NCBI nr library, Swissprot database, Kyoto Encyclopedia of Genes and Genomes (KEGG) database, Ensembl zebrafish database, and Ensembl tilapia database using NCBI-blast-2.5.0, with a threshold of 1e-05. We used the orthology module in Ensembl biomart to obtain the human homologs of genes annotated to zebrafish. In this project, marker genes were compiled, and a featurePlot was plotted to visualize gene expression distribution and identify clusters. The heatmap clearly shows the expression of known marker genes in different clusters. After the annotation was completed, the cell types obtained from the annotation were mapped to UMAP and tSNE maps.

### 2.6 Differential and functional enrichment analysis

For each cluster, genes with expression that differed between sample sources were analyzed, and the threshold for TAV vs. CK differential gene screening was │log2(FC) │ > 0.25 and *p* < 0.05. The differential genes of each cluster of each sample were subjected to KEGG (Kyoto Encyclopedia of Genes and Genomes) and GO (Gene Ontology) enrichment analysis. GO enrichment analysis is an international standardized transcriptional function classification system that provides a set of dynamically updated standard taxonomic names to adequately describe the properties of transcripts and transcript products in organisms (Falcon and Gentleman, 2007). GO function analysis provides taxonomic annotation of differentially expressed transcripts as well as a significant enrichment analysis of differentially expressed transcripts (Kanehisa and Goto, 2000).

### 2.7 Granulocyte cell and B cell subpopulation analysis

#### 2.7.1 Subtype analysis

Granulocyte cells and B cells were analyzed in further detail. The subpopulations of cells were separated out and then re-dimensioned, clustered, and annotated. After the annotation was completed, the annotated types were mapped to the UMAP and tSNE maps.

## 3 Results

### 3.1 Dimensionality reduction and clustering results

**FIGURE 1:**
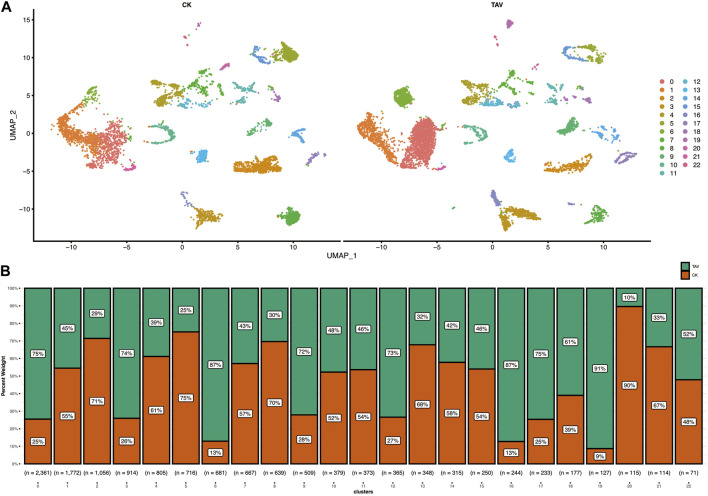
**(A)** Twenty-two cell clusters with uniform flow shape approximation and projection (UMAP) space were identified and displayed. **(B)** Statistical histogram showing the number of cells in each cluster for the TAV and CK groups.

Cell viability was confirmed to be approximately 98% by microscopic examination. The total number of cells measured in the total sample was 13,382, with 7174 cells detected in the TAV group and 6208 cells detected in the CK group. After quality control and mapping using Cell Ranger software, the 13,382 cells had a total read length of 589,228,107 bp with an average read length of 44,031 bp per cell acquisition (Figure 1). In total, 23 cell clusters (clusters 0–22) were characterized (Figure 1A). The percentage of each cell cluster in the TAV and CK groups is shown in Figure 1B.

### 3.2 Cell subpopulation identification results

**FIGURE 2:**
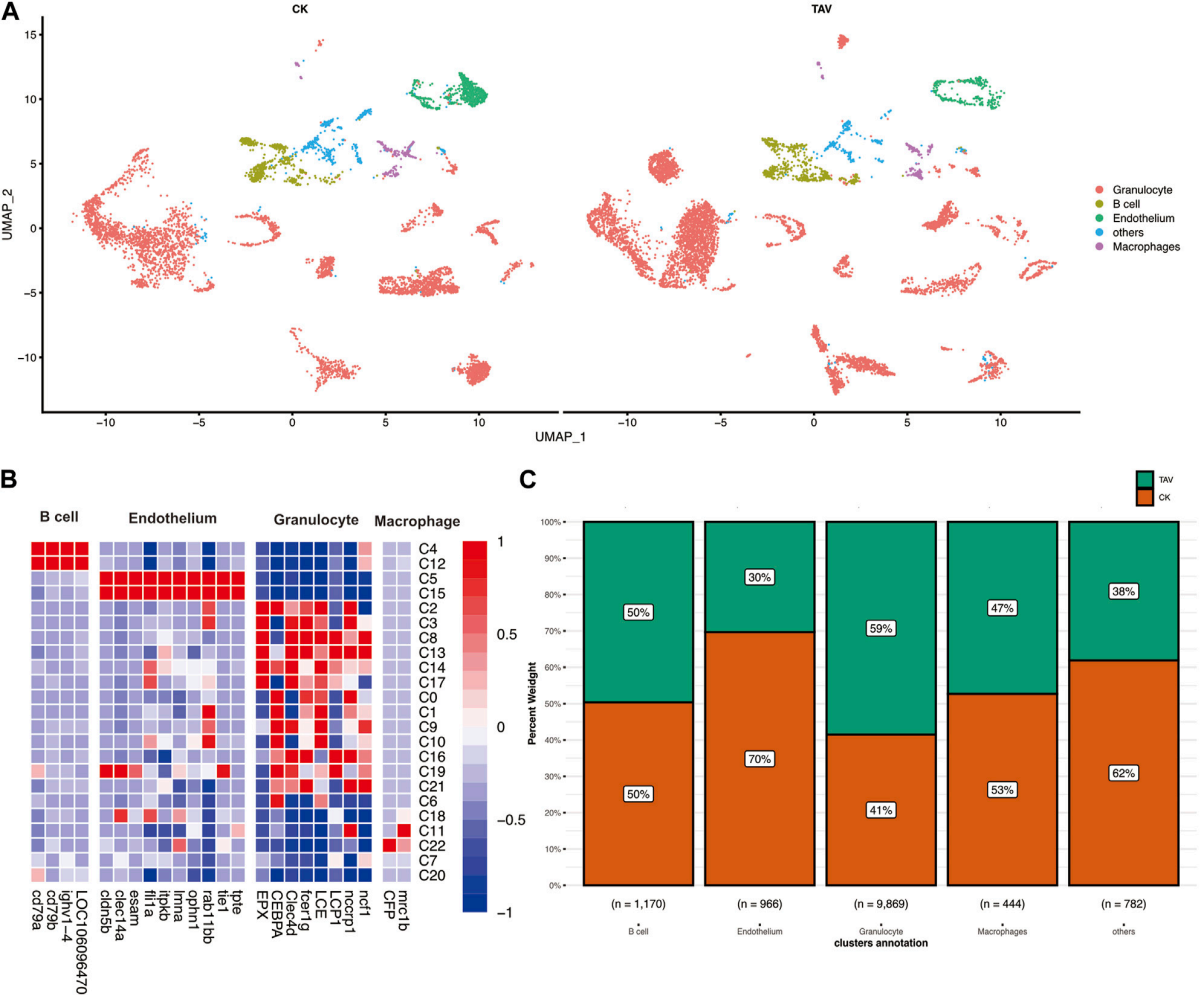
**(A)** UMAP plot showing identification of putative cell types based on the expression of marker genes in mammals and fish. **(B)** Heatmap of putative marker genes in B cells, endothelial cells, macrophages, and granulocytes in cell clusters, with the expression levels of genes in different cells indicated by different colors. The redder the color, the higher the expression level, and the more purple the color, the lower the expression level. **(C)** Histogram showing the number of cells per cell type for B cells, endothelial cells, macrophages, and granulocytes.

Twenty-three cell clusters (clusters 0–22) were characterized (Figure 2A). Each cell subset-specific gene is shown in the heatmap, and the 23 cell clusters were grouped into B cells, granulocytes, endothelial cells, and macrophages (Figure 2B). In the TAV group, 5774 cells from cell clusters 0, 1, 2, 3, 6, 8, 9, 10, 13, 14, 16, 17, 18, 19, and 21; and 4095 cells from the CK group were classified as granulocytes. In the CK group, 589 cells in cell clusters 4 and 12 and 581 cells from the TAV group were classified as B cells. In the CK group, 234 cells from clusters 11 and 22, and 210 cells from the TAV group, were classified as macrophages. The remaining 484 cells in the CK group and 298 cells in the TAV group were not identified but were found in clusters 7 and 20. These results show a relatively large difference between granulocyte and endothelial cell numbers between the TAV and CK groups. Granulocytes as a whole were more abundant in the TAV group, whereas endothelial cells as were less abundant in the TAV group, compared to the CK group (Figure 2C).

### 3.3 Granulocyte subpopulation analysis results

**FIGURE 3:**
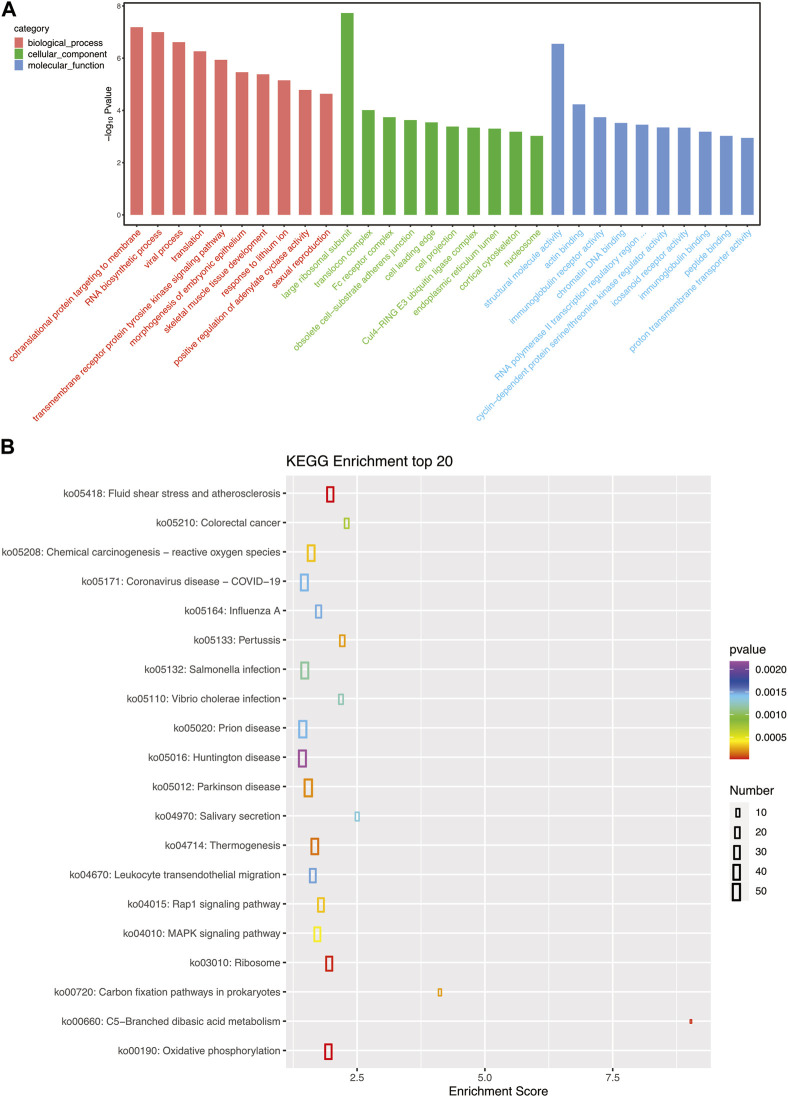
**(A)** GO pathways enriched for each granulocyte subset gene; the top 10 most important GO pathways enriched in cellular processes, molecular functions, and cellular components are shown. **(B)** The top 20 most important KEGG pathways enriched for each granulocyte subset gene.

To examine the biological functions of DEGs, GO and KEGG pathway analysis was performed on all DEGs. GO annotations of these genes were classified into three categories based on their functions and pathways: biological processes, cellular components, and molecular functions. Large ribosomal subunit in cellular components and structural molecular activity in molecular functions were the significantly influenced functions (Figure 3A). KEGG pathway analysis also demonstrated the influence of *A. veronii* on related pathways (Figure 3B). Pathways significantly affected by the bacterium were ribosome (ko03010), fluid shear stress and atherosclerosis (ko05418), and oxidative phosphorylation (ko00190).

**FIGURE 4:**
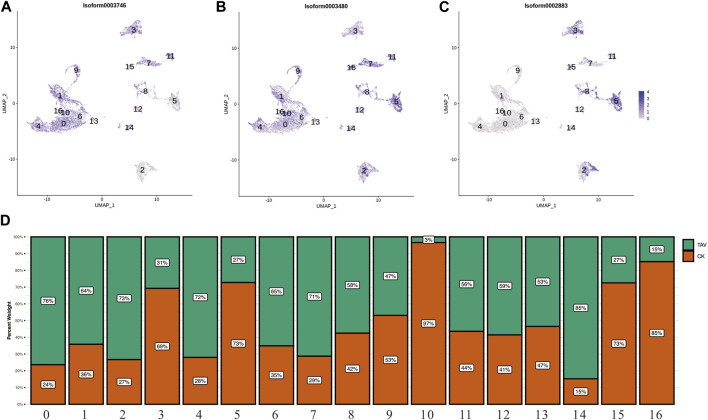
**(A)** UMAP plots of the neutrophil marker gene CEBPA (Isoform0003746). **(B)** UMAP plots of the neutrophil marker gene ncf1 (Isoform0003480). **(C)** UMAP plots of the eosinophil marker gene EPX (Isoform0002883). **(D)** Histogram showing the number of cells per cell cluster in granulocytes.

Neutrophil marker genes CEBPA (Isoform0003746), ncf1 (Isoform0003480), EPX (Isoform0002883) were observed in UMAP (Figures 4A–C). Neutrophils were present in the cell clusters 1, 4, 6, 9, 10, 13, 14, and 16; and eosinophils were present in cluster 5 (Figure 4). Relative to the control (TAV), neutrophil number was significantly higher and eosinophil number was significantly lower in the granulocytes in the CK group (Figure 4D).

### 3.4 Results of B cell subpopulation analysis

**FIGURE 5:**
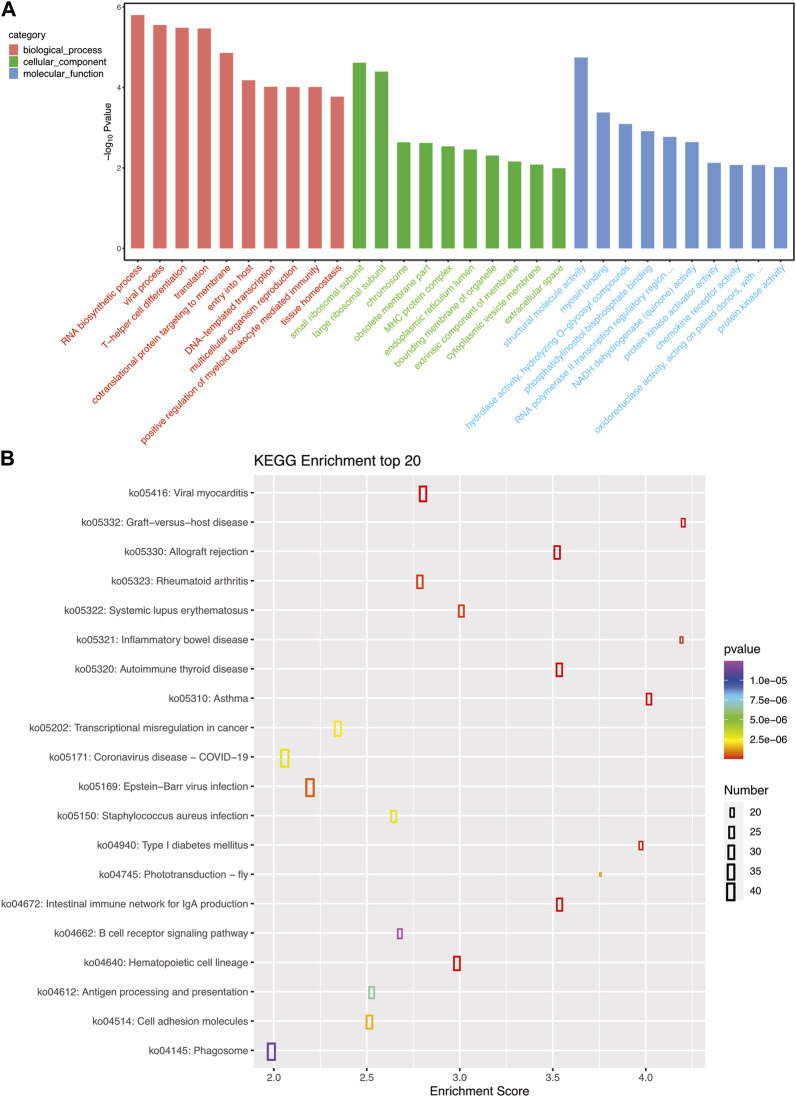
**(A)** GO pathways enriched for each B cell subset gene; the top 10 most important GO pathways enriched in cellular processes, molecular functions, and cellular components are shown. **(B)** The top 20 most important KEGG pathways enriched for each B cell subset gene.

As shown in Figure 5A, the biological processes in the GO pathway that were significantly affected by bacterial infection were RNA biosynthesis process, viral process, and T helper cell differentiation. In the cellular components category, the ribosomal small subunit and ribosomal large subunit were the significantly affected functions. The structural molecular activity in the molecular functions category was the significantly affected function, which is consistent with the affected pathways identified in granulocytes. The KEGG pathway analysis showed that bacterial infection significantly affected immune relative pathway such as graft-versus-host disease (ko05332), viral myocarditis (ko05416), and inflammatory bowel disease (ko05321) pathways (Figure 5B).

**FIGURE 6:**
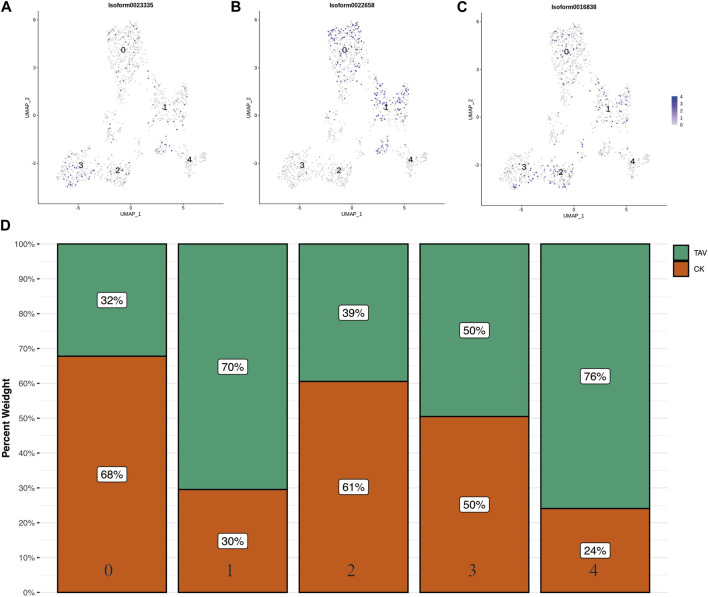
UMAP plots of B cell universal marker gene **(A)** *cd79a* (Isoform0023335), **(B)** *cd79b* (Isoform0022658), and **(C)** *ighv1-4* (Isoform0016838). **(D)** Histograms of the number of cells per cell cluster type in B cells.

The UMAP of B cell marker genes *cd79a* (Isoform0023335), *cd79b* (Isoform0022658), and *ighv1–4* (Isoform0016838) were obtained based on relevant literature and transcriptome data (Figures 6A–C). Cell clusters 0, 1, 2, 3, and 4 could not be identified based on the available B cell marker genes. The number of cells in clusters 1 and 4 in B cells was significantly higher in the TAV group compared to the CK group (Figure 6D).

## 4 Discussion

Research on markers of teleost cell populations and cell subpopulation determinants is still relatively sparse. However, several cutting-edge methods, including scRNA-seq, are now being used to study the cellular immunological functions of fish. In this study, we used scRNA-seq to investigate the effects of infection with *A. veronii* on the head kidney cells of *O. potamophila* and on the regulation of gene expression.

In this study, cells from *O. potamophila* were categorized into B cells, endothelium cells, macrophages, and granulocytes based on the expression patterns of marker genes. However, we were unable to identify the full range of cellular subpopulations based on known marker genes. The differentiation or polarization of these immune cells (e.g., macrophages can polarize into M1 or M2 macrophages) is triggered by corresponding cytokines and transcription factors in response to stress and immune and inflammatory responses (Uribe et al., 2011). Not all cell subtypes were present in the kidneys of *O. potamophila* and other single-cell sequencing studies of cells isolated from fish kidney tissue did not find rag1 gene expressing cells (i.e., these fish lack mature T cells), which may explain the inability to identify these cell subtypes (Wang et al., 1996; Moore et al., 2016). However, the cell clusters obtained in this study will provide useful information for further research of the inflammatory response of *O. potamophila*. We used UMAP analysis to visualize transcriptional differences between B cell subpopulations, but the results showed that marker genes were not significantly different among different subpopulations of B cells (Figures 6A–C). This result likely reflects the widespread and unpredictable heterogeneity in the expression of these marker genes across cells.

In granulocyte clusters, eosinophil protein X (*EPX*) is a specific marker gene for eosinophils (Wechsler et al., 2021) and CLECSF8 (*CLEC4D*) (Wilson et al., 2015), CCAAT/enhancer-binding protein-alpha (*CEBPA*), and ecnccrp-1 (*NCCRP1*) (Ishimoto et al., 2004) are neutrophil-specific marker genes. Members of the Cebp family are well-known key regulators involved in neutrophil development, and *CEBPA* plays a key role in the proliferation of mitotic neutrophil progenitor cells (Zhang et al., 1997; Xie et al., 2020). Granulocytes are known to contain neutrophils, eosinophils, basophils, and mast cells (Ainsworth, 1992). Our results showed that neutrophil numbers in granulocytes increased significantly after fish were infected with *A. veronii*, whereas eosinophil numbers decreased. Neutrophils migrate from the circulation to infected tissues in response to inflammatory stimuli and protect the host by phagocytosing, killing, and digesting bacterial and fungal pathogens (Newburger, 2006; Amulic et al., 2012). Significant enrichment of ribosomal biogenesis was detected by GO analysis, indicated that genes related to structural molecular activity and large ribosomal subunit were affected by bacterial infection. The protein components, also known as ribosomal proteins (rps), play a critical role in ribosome and protein synthesis, and several perform important extra-ribosomal functions and are involved in DNA repair, transcriptional regulation, and apoptosis (Chang et al., 2015). Expression of RPS12 and RPL12 were upregulated after infection with *A. veronii*, which indicated more protein synthesis in cells (Supplementary material S1). This results in a highly functional cell population, with neutrophils activated to increase ribosomal protein levels to fight against invading pathogens (Schneider et al., 2014).

Fish B cells are functioning antibody-secreting cells that generate particular antibodies in response to external invader antigens, and they are crucial for adaptive immunity (Parra et al., 2013). Unlike mammals, there are no specific antibodies that can be used to accurately distinguish the developmental/differentiation status of fish B cells, which hinders studies of their function. In the present study, clusters of cells expressing cd79a (Minegishi et al., 1999), cd79b (Niu et al., 2020), and ighv1-4 (Tang et al., 2017) were identified as B cell populations. *CD79b* and *CD79a* are genes that encode the B cell receptor accessory proteins B29 and mb1 (Huse et al., 2022). The IGHV1-4 expression product, immunoglobulin M (IgM) is thought to be a ubiquitous vertebrate immunoglobulin that innately recognizes and binds a variety of antigens (Dooley and Flajnik, 2005). IgM has been used as a marker of mature B cells in trout and grouper (Zhang et al., 2010; Castro et al., 2014). IgM+ B cells have a strong phagocytic capacity and are able to kill microorganisms that are phagocytosed by the cells (Li et al., 2006). Subsequent studies have shown that rainbow trout IgT + B cells also contain subpopulations with phagocytic and bactericidal capabilities (Zhang et al., 2010). Other teleost species, including catfish, cod, and Atlantic salmon, contain phagocytic B cells and feature adaptive immune responses to characteristic pathogens (Øverland et al., 2010).

GO enrichment analysis of DEGs in B cells from fish infected with *A. veronii*, RNA biosynthesis process, viral process, and T helper cell differentiation significant changes. Expression of crucial pro-inflammatory mediators such as *IL1B* and *IGHV1-4*, which are involved in virulence processes, was upregulated (Supplementary material S1), suggesting that *A. veronii* activates immunoglobulin receptors. Major histocompatibility complex (MHC) class II genes *MHC2A* and *MHC2DAB* were also upregulated after infection with *A. veronii.* Antigen-presenting cells are key regulators of immunity, and the expression of MHCII molecules is restricted to some of them, including B cells (Watts, 1997). B cells utilize the specialized MHCII antigen presentation pathway to process B cell receptor-bound and internalized protein antigens and then present selected peptides in complex with MHCII to CD4^+^ T cells. The immune response of B cells of *O. potamophila* stimulated by *A. veronii* was similar to that of mammalian B-1 B cells, with IgT^+^ and IgM^+^ head kidney B cells proliferating rapidly and secreting IgT and IgM, respectively, in response to pathogenic stimulation (Zhang et al., 2010).

## 5 Conclusion

In this study, we used the expression of marker genes to group *O. potamophila* cells into B cells, endothelial cells, macrophages, and granulocytes, and we performed differential enrichment analysis of gene expression in B cells and granulocytes of fish infected with *A. veronii*. The combined analysis revealed a significant increase in neutrophils and decrease in eosinophils in granulocytes of fish infected with *A. veronii*. Activation of neutrophils enhanced ribosome biogenesis by up-regulating the expression of *RPS12* and *RPL12* to fight against invading pathogens. Crucial pro-inflammatory mediators such as *IL1B, IGHV1–4*, and MHC class II genes *MHC2A* and *MHC2DAB*, which are involved in virulence processes, were upregulated, suggesting that *A. veronii* activates an immune response that presents antigens and activates immunoglobulin receptors in B cells. These cellular immune responses identified by single-cell sequencing increase our knowledge about teleost species and lay the foundation for subsequent cellular immune studies.

## Data Availability

The datasets presented in this study can be found in online repositories. The names of the repository/repositories is NCBI and accession number(s) is GSE229275.
