# Supplementary material for: Single-cell transcriptome analysis reveals a cellular immune response in freshwater dark sleeper (Odontobutis potamophila) after infection with Aeromonas veronii
Source: Front Physiol. 2023 May 18;14:1201914. doi: 10.3389/fphys.2023.1201914 (PMC10233152; doi:10.3389/fphys.2023.1201914)
Supplement: Supplementary file 1 [file Table1.DOCX]

Supplementary Material

Article Title

**Guoxing Liu^1,2,3†^, Chenxi Zhu^2†^, Xiaojian Gao^1^, You Zheng^2,3^, Xinhai Zhu^1^, Hucheng Jiang^2,3^, Wanhong Wei^1^, Qichen Jiang^1,2,3*^ and Xiaojun Zhang^1*^**

*** Correspondence:** Qichen Jiang, Xiaojun Zhang

qichenjiang@live.cn, [zxj9307@163.com](mailto:zxj9307@163.com)

Supplementary Data

# Supplementary Figures and Tables

**Table 1.** Significant changes in immune genes in cells

| id | p_val | avg_log2FC | id | Dre_Gene_ID | Dre_Gene_symbol | cell type |
| --- | --- | --- | --- | --- | --- | --- |
| Isoform0026450 | 0 | 2.7689434 | Isoform0026450 | ENSDARG00000006691 | rpl12; | neutrophils |
| Isoform0027678 | 0 | 1.790202784 | Isoform0027678 | ENSDARG00000115157 | rps12; | neutrophils |
| Isoform0023145 | 4.08E-07 | 1.058215651 | Isoform0023145 | ENSDARG00000098700 | il1b; | B cells |
| Isoform0025430 | 0.00047342 | 0.89393189 | Isoform0025430 | ENSDARG00000031745 | mhc2a; | B cells |
| Isoform0023662 | 0.00049029 | 0.743168096 | Isoform0023662 | ENSDARG00000096355 | ighv1-4; | B cells |
| Isoform0011698 | 1.60E-11 | 1.093179561 | Isoform0011698 | ENSDARG00000079105 | mhc2dab; | B cells |
